# Acute interaction between hydrocortisone and insulin alters the plasma metabolome in humans

**DOI:** 10.1038/s41598-017-10200-9

**Published:** 2017-09-13

**Authors:** Mohammad A. Alwashih, Roland H. Stimson, Ruth Andrew, Brian R. Walker, David G. Watson

**Affiliations:** 1 Strathclyde Institute of Pharmacy and Biomedical Sciences, University of Strathclyde, Glasgow, G4 0RE UK; 2 BHF Centre for Cardiovascular Science, Queen’s Medical Research Institute, University of Edinburgh, Scotland, UK; 3 General Directorate of Medical Services, Ministry of Interior, Riyadh, 13321 Saudi Arabia

## Abstract

With the aim of identifying biomarkers of glucocorticoid action and their relationship with biomarkers of insulin action, metabolomic profiling was carried out in plasma samples from twenty healthy men who were administered either a low or medium dose insulin infusion (n = 10 each group). In addition, all subjects were given metyrapone (to inhibit adrenal cortisol secretion) + /− hydrocortisone (HC) in a randomised crossover design to produce low, medium and high glucocorticoid levels. The clearest effects of insulin were to reduce plasma levels of the branched chain amino acids (BCAs) leucine/isoleucine and their deaminated metabolites, and lowered free fatty acids and acylcarnitines. The highest dose of hydrocortisone increased plasma BCAs in both insulin groups but increased free fatty acids only in the high insulin group, however hydrocortisone did not affect the levels of acyl carnitines in either group. The clearest interaction between HC and insulin was that hydrocortisone produced an elevation in levels of BCAs and their metabolites which were lowered by insulin. The direct modulation of BCAs by glucocorticoids and insulin may provide the basis for improved *in vivo* monitoring of glucocorticoid and insulin action.

## Introduction

Intracellular glucocorticoid receptors are widely expressed and affect energy metabolism, cardiovascular control and innate immunity^1–5^. Acute elevation in cortisol is a crucial component of the stress response, but chronic glucocorticoid excess (Cushing’s syndrome) causes obesity, type 2 diabetes, hypertension, impaired immunity, depression, and cognitive dysfunction. Common medical conditions such as type two diabetes and the metabolic syndrome^3^ and neuropsychiatric disease^4^ are associated with mildly elevated circulating and tissue glucocorticoid levels although at present it is not possible to measure circulating markers of tissue glucocorticoid action. Similarly, glucocorticoid deficiency, which can be life-threatening during stress, is especially difficult to diagnose during critical illness when conventional tests of cortisol production such as adrenocorticotrophic hormone (ACTH) stimulation tests may be unreliable^6^. This lack of specific biomarkers makes clinical management of patients requiring glucocorticoid replacement therapy particularly challenging, and may contribute to well-documented excess morbidity and mortality in patients with hypopituitarism or adrenocortical failure^7–10^. The complexity of glucocorticoid action imposes a major limitation in the development of new therapeutic agents, because of the lack of reliable indicators of a reduction in tissue cortisol action.

Novel biomarkers of glucocorticoid action are urgently required and mass spectrometry has become increasingly applied to this area. A previous study using gas chromatography with mass spectrometry (GC-MS) reported limited metabolomic profiling in urine and plasma of subjects treated with anti-inflammatory synthetic glucocorticoids^11^. A recent more comprehensive study was carried out using GC-MS and liquid chromatography-mass spectrometry (LC-MS) to both profile and quantify the metabolome of 20 healthy male volunteers following administration of the synthetic glucocorticoid dexamethasone^12^, which reduced plasma levels of alanine, methionine, asparagine, phenylalanine, proline and serine.

Comparison of groups of people found a number of markers to be altered in insulin resistant individuals, particularly unsaturated fatty acids^13^. A distinct metabolic signature has been linked to obesity: elevated plasma levels of the branched chain amino acids (BCAs) leucine, isoleucine and valine; elevated amino acids methionine, glutamine, phenylalanine, tyrosine, asparagine, and arginine, with concomitant depression of glycine levels^14^; and elevations of a number of free fatty acids and acyl carnitines. In fact, it has been proposed that BCA levels provide a better signature of metabolic health than BMI^15^ and may predict development of insulin resistance^16^.

To date, no direct study on the effects on insulin and cortisol on metabolic profiles in individuals has been reported. To identify biomarkers which reflect glucocorticoid and insulin action, we performed metabolomic analysis of plasma samples obtained from a previously published study of healthy men treated with metyrapone followed by hydrocortisone (HC) infusion to induce low (~150 nM), medium (~400 nM) and high (~1400 nM, supra-physiological) circulating cortisol levels^17^. Moreover, we tested specificity of the response to glucocorticoids by making measurements before and after insulin infusion, and examined the interaction between insulin and HC.

## Results

### Quality control

**Figure 1:**
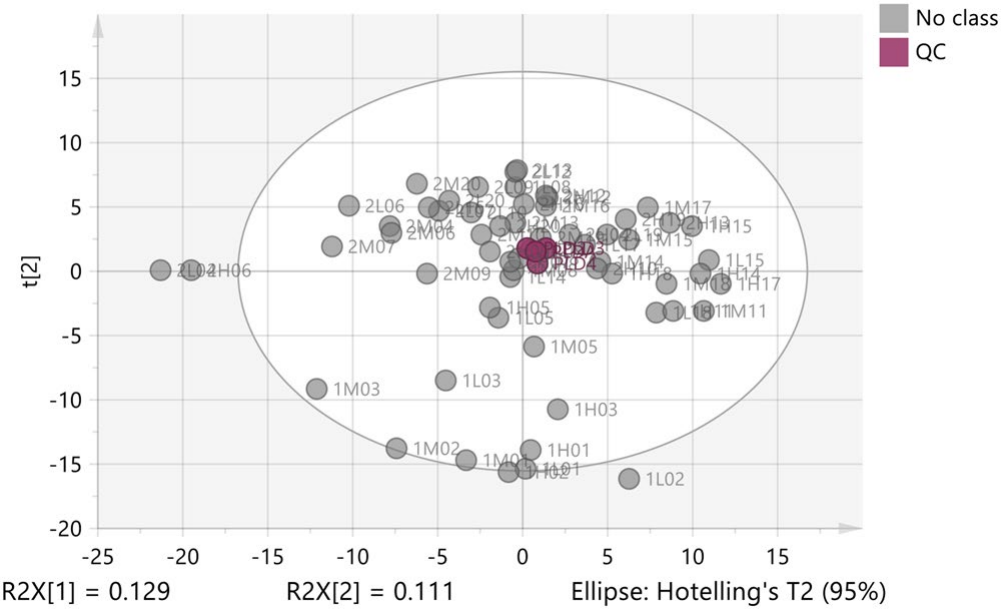
2D PCA score plot for QC (pooled) samples in healthy individuals. The plot shows the clustering of pooled samples (plum-QC) compared to the rest of plasma samples (grey-No class).

Metabolomic profiling of subjects was carried out by using LC-MS. A pooled plasma sample was prepared and the instrument was set to inject the pooled sample after every 15 plasma samples, thus the pooled sample had 4 readings (Fig. 1). To quantify the precision of the measurements, the relative standard deviation (RSD) was calculated between the 4 pooled samples based on the sum of the intensities in each sample and an RSD of 0.5% was obtained. The RSD was also calculated for each of the metabolites in the pooled samples and the highest RSD was for 38:6 glycero-3-phosphoethanolamine (9.7%) while the lowest RSD was for alanine (0.23%). The precision of these values clearly indicates that any metabolomic differences between groups could not be due to instrumental factors alone.

### Data visualization

**Figure 2:**
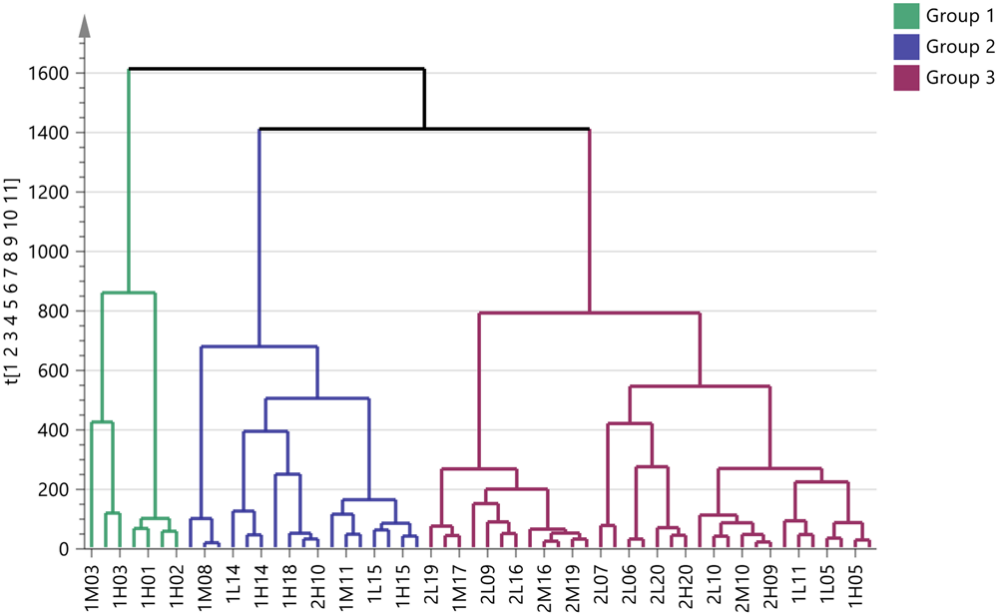
Hierarchical Clustering Analysis (HCA). The dendrogram above shows observations clustered into three groups. X-axis represents the samples and y-axis shows the similarity index. The higher the variability index the larger the between group variability and the lower the similarity index, the smaller the between group variability. The plot divides samples into three groups; group 1 (green), group 2 (blue) and group 3 (plum).

A hierarchical clustering analysis (Fig. 2) shows that samples almost clustered according to the insulin dose, group 3 consists of 32 samples, about 80% of the samples are for individuals receiving a high insulin dose. 100% of the observations in group 1, and also about 89% of the observations in group 2 are for individuals receiving a low insulin dose (Table 1). However, there were no significant differences in HC dose between groups (Table 1). Thus the HC doses did not show as large a contribution to the clustering pattern in comparison to the insulin dose.

**Table 1:** Proportions of HC and insulin doses based on the HCA grouping.

HCA grouping	Observations (n)	Percentage in each group (%)					
		Low Insulin			High Insulin		
		Low HC	Medium HC	High HC	Low HC	Medium HC	High HC
1 (Green)	7	28.6	28.6	42.9	0.0	0.0	0.0
2 (Blue)	17	23.5	29.4	35.3	0.0	0.0	11.8
3 (Plum)	32	9.4	6.3	3.1	28.1	31.3	21.9

**Figure 3:**
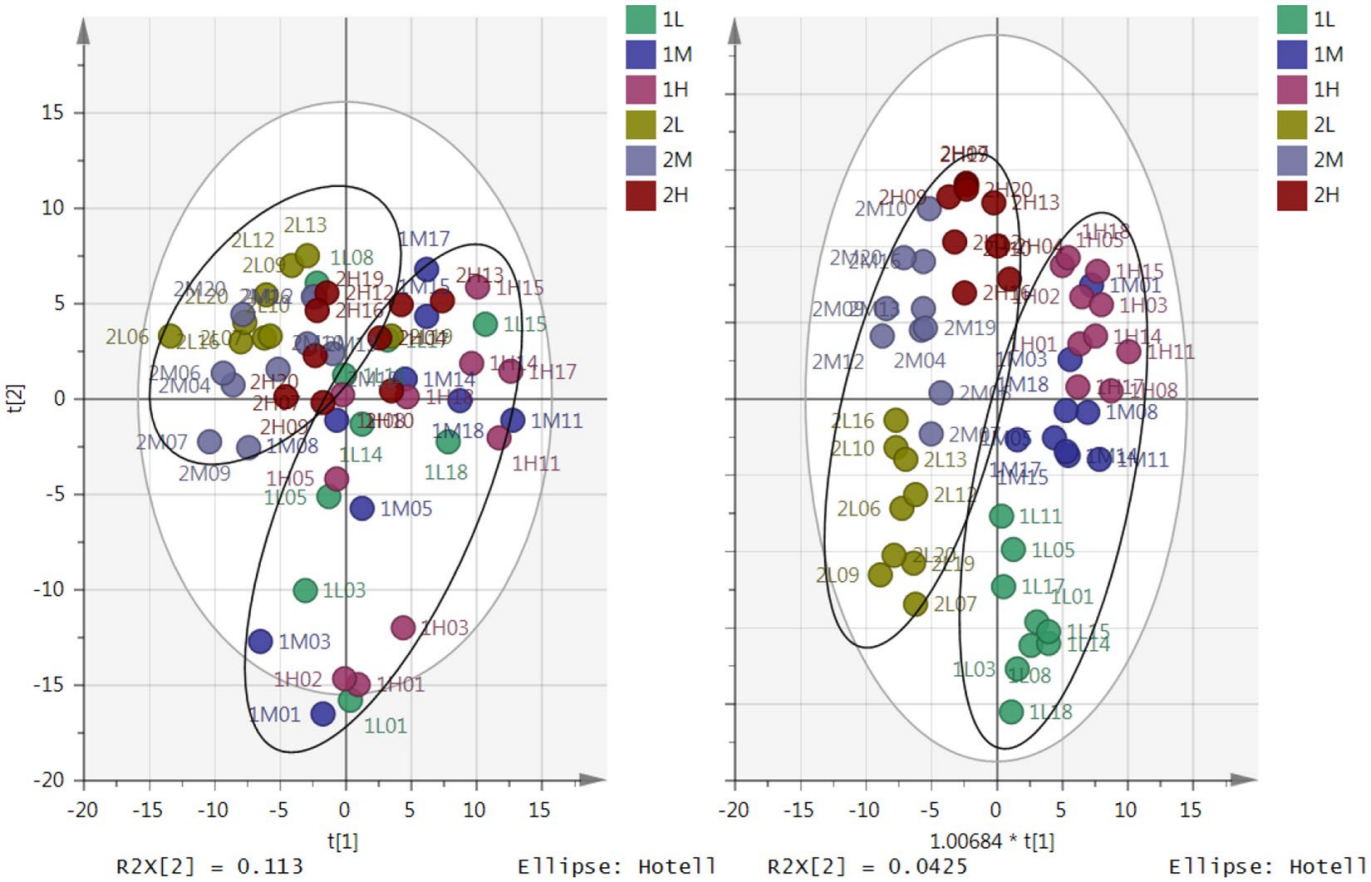
(**A**, left) PCA vs (**B**, right) OPLS-DA score plots for healthy individuals receiving different doses of HC and insulin. PCA score plot (**A**) includes 2 groups of subjects (n = 30 samples/10 subjects/group). Group 1 denotes samples with low insulin dose (n = 30), group 2 denotes samples with high insulin dose (n = 30). Subjects in each group have 3 different levels of HC treatment; L = low HC, M = medium HC and H = high HC dose. OPLS-DA score plot (**B**) includes the same group of subjects. Subjects in the same oval shapes were given the same insulin dose. In the OPLS-DA, model separation is between low and high HC doses in each insulin group but the domain of the medium HC dose overlaps with that of high HC dose in both insulin groups, while in the high insulin group also overlaps with the low GC dose.

A principal components analysis (PCA) score plot (Fig. 3A) showed separation between subjects having the highest insulin dose from those receiving a low insulin dose. However, subjects with the highest insulin dose also tended to show separation based on the HC dose. In addition, it is noticeable that subjects receiving a high HC dose overlap to some extent with the low insulin group and conversely there is some overlap between low HC and high insulin.

Separation between groups based on both insulin and HC dose can be seen more clearly in the OPLS-DA score plot (Fig. 3B), where the analysis is supervised, although subjects receiving medium and high HC dose showed some overlap where a low insulin dose was given.

### The effect of insulin on mtabolomic changes in plasma

**Figure 4:**
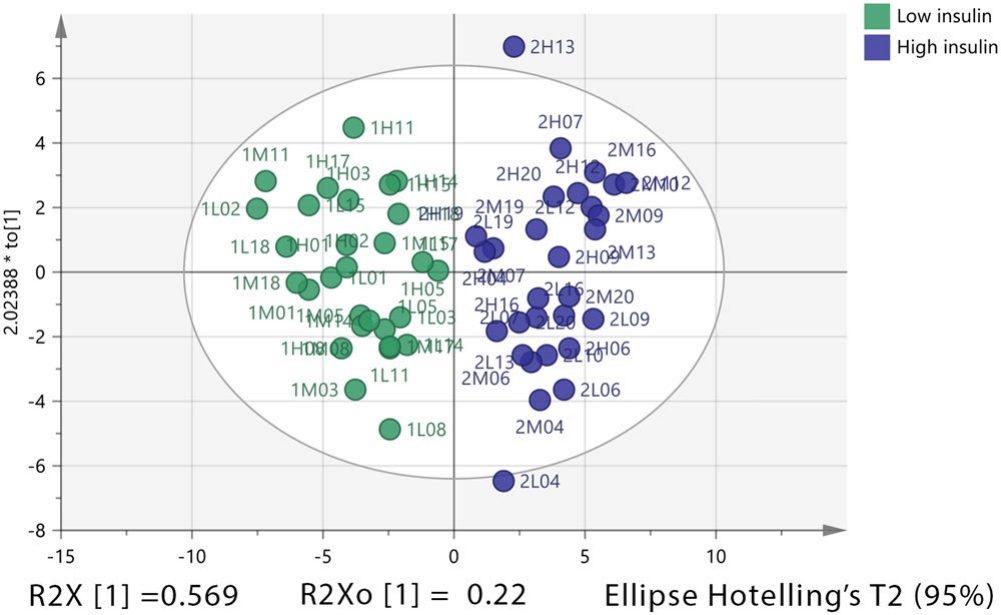
OPLS-DA score plot for healthy individuals having either high or low insulin dose. The OPLS-DA score plot shows two groups of samples (n = 29 samples per group) based on readings of 29 significant metabolites in plasma of healthy individuals. Subjects with low insulin dose (green) and subjects with high insulin dose (blue). The model consists of one predictive x-score component; component t[1] and one orthogonal x-score components to[1]. t[1] explains 56.9% of the predictive variation in x, to[1] explains 22% of the orthogonal variation in x, R^2^X (cum) = 0.789, R^2^Y (cum) = 1, R^2^ (cum) = 0.841, Accuracy of prediction Q^2^ (cum) = 0.796.

An OPLS-DA model was built on 606 features, which were detected in 60 observations having either low or high insulin dose. Two observations (1M02 and 2H10) were excluded as they were considered to be outliers based on Hotellings’T^2^ vs DModX plot, leaving 58 observations (29 observations per group) from the high and low insulin groups. The resulting model identified 31 putative biomarkers (Table 2) which were selected based on their 95% confidence interval of difference (CI), corrected p-values ( < 0.05), and the AUC of their ROC curves ( > 0.7). These metabolites were then used to rebuild the OPLS-DA model (Fig. 4) in order to examine its ability to separate the subjects based on the insulin dose.

**Table 2:** Metabolites significantly altered by insulin dose.

Metabolite	AUC	High insulin/Low insulin	*p*-value
**Polyunsaturated fatty acids**			
C22:4	0.87	0.48	0.0001
C22:6	0.86	0.46	0.0001
C20:2	0.92	0.37	0.000005
C20:4	0.81	0.59	0.002
C18:2	0.94	0.29	6.38E-07
C18:3	0.94	0.27	0.000002
**Monounsaturated fatty acids**			
C20:1	0.97	0.26	4.40E-07
C18:1	0.94	0.30	0.000003
C16:1	0.95	0.23	6.68E-07
C14:1	0.90	0.32	0.00001
**Straight chain fatty acids**			
C20:0	0.86	0.53	0.0001
C17:0	0.88	0.44	0.00001
C16:0	0.92	0.36	0.000007
C15:0	0.88	0.45	0.0001
C14:0	0.89	0.35	0.00002
C10:0	0.84	0.60	0.001
**Acyl carnitines**			
O-Acetylcarnitine*	0.92	0.57	4.60E-07
Decanoylcarnitine	0.91	0.47	0.000002
Oleoylcarnitine	0.96	0.58	0.000007
**Branched chain amino acids**			
L-Leucine*	0.79	0.74	0.003
3-Methyl-2-oxopentanoic acid^C18^	0.77	0.71	0.001
4-Methyl-2-oxopentanoate*	0.75	0.67	0.003
L-Isoleucine*	0.82	0.76	0.00008
L-Valine*	0.77	0.85	0.021
**Miscellaneous**			
Hydroxybutanoic acid	0.93	0.28	0.00006
2-Hydroxybutanoic acid*	0.73	0.64	0.013
Indolepyruvate	0.79	1.34	0.008
Gamma-Glutamylglutamine	0.73	0.81	0.017
Glycerol	0.90	0.74	0.0001

*Retention time confirmed by standard. ^C18^ metabolites identified using C18-AR column, the rest identified using ZICpHILIC column. (L = low insulin dose, H = high insulin dose), p-value is an output of split-plot ANOVA.

The OPLS-DA score plot (Fig. 4) showed clear separation between the two groups; around 79% of the variability in metabolites was explained by the model, of which 57% was due to insulin dose alone with the rest being attributable to systemic or orthogonal variations. 84% of the variability between the observations was explained by the variability in the metabolites, of which 80% was predicted by the model following cross-validation. The validity of the number of orthogonal components in the model was examined using observed versus predicted plot. The regression line in the plot had R^2^ = 0.84 (Figure S1A, supplementary) indicating acceptable model validity. Based on a permutation test (Figure S1B, supplementary) this model had a valid predictive ability compared to the newly permuted Q^2^ values. Using 31 putative metabolites that were significantly changed by the insulin dose it was possible to classify 100% of the observations based on the insulin dose (Figure S2).

Table 2 shows that all the marker metabolites were significantly decreased (p < 0.05) at high compared to low insulin doses except for indolepyruvate which was significantly increased (p = 0.008). The majority of these metabolites demonstrated excellent classifying ability based on their AUC values (AUC > 0.9). Of the metabolites which were separated on the C18 column, only 3-Methyl-2-oxopentanoic acid was significantly affected (H/L ratio = 0.71; p = 0.001) although it only had a moderate classifying ability (AUC = 0.77)^18^.

**Figure 5:**
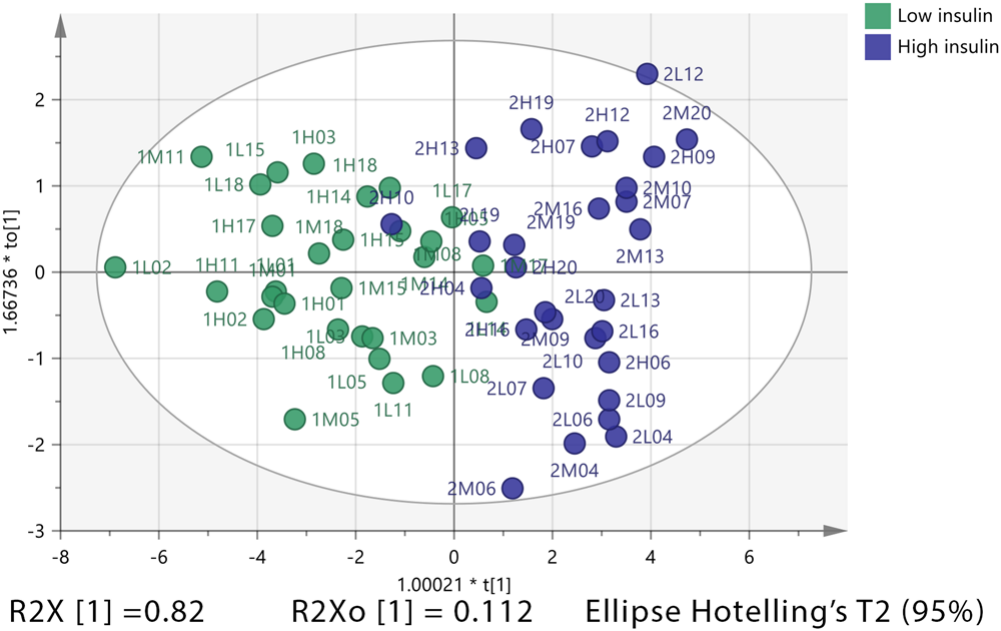
OPLS-DA score plot for the effect of insulin on 10 selected metabolites. The OPLS-DA score plot based on 10 most significant metabolites showing two groups: samples with low insulin dose (green) and samples with high insulin dose (blue). The model consists of one predictive x-score component; component t[1] and one orthogonal x-score components to[1]. t[1] explains 82% of the predictive variation in x, to[1] explains 11.2% of the orthogonal variation in x, R^2^X (cum) = 1, R^2^Y (cum) = 1, R^2^ (cum) = 0.706. Accuracy of prediction Q^2^ (cum) = 0.665.

Generally, in order to avoid the possibility of over-fitting in an OPLS-DA model, the number of the variables should be less than the total number of observations. Xia *et al*. suggested that using 1–10 biomarkers for classification is more statistically robust and clinically more practical^18^. An OPLS-DA model (Fig. 5) was built on 10 variables having the highest AUC values among the metabolites significantly affected by the insulin dose (Table 3) in plasma samples of 58 subjects (low insulin = 29, high insulin = 29). All these metabolites were strongly negatively correlated to insulin dose (|r| > 0.88). Two observations (1M02 and 2M12) were excluded as they were strong outliers based on Hotelling’sT^2^ vs DModX plot. The model shows that approximately 93% of the variations in these putative biomarkers were explained by the model; 82% of this variation was due to the insulin dose with *P* CV-ANOVA = 4.91E-12, while approximately 12% was due to inter-individual variability. The AUC shows an excellent ability of these metabolites to classify 98% of the subjects based on insulin dose using 10 biomarkers.

**Table 3:** The 10 metabolites with highest AUC values and their correlations (r) to insulin dose.

Putative biomarkers	r	99% CI of difference
C20:2	−0.88	(−0.301, −0.227)
C18:3	−0.92	(−0.297, −0.254)
C18:2	−0.92	(−0.3, −0.254)
C18:1	−0.94	(−0.304, −0.259)
C16:1	−0.92	(−0.285, −0.267)
C20:1	−0.94	(−0.318, −0.25)
Hydroxybutanoic acid	−0.83	(−0.316, −0.181)
Palmitic acid	−0.89	(−0.3, −0.234)
O-Acetylcarnitine	−0.88	(−0.33, −0.194)
Oleoylcarnitine	−0.94	(−0.367, −0.198)

### Effect of hydrocortisone on the plasma metabolome

A total of 606 putative biomarkers were measured in 60 subjects receiving 3 different HC doses: low HC dose = 20 subjects, medium dose = 20 subjects and high dose = 20 subjects. 4 observations (1L02, 1M02, 2L04 and 2H06) were excluded as they were considered to be outliers based on Hotellings’T^2^ vs DModX plot. The medium HC dose was found to be a poorly classified by the biomarkers having AUC of 0.67 compared to low and high HC dose (AUC = both 1.0), and thus had a high proportion of misclassified observations (47.4%) compared to the other two doses (0% each) (Table S1, supplementary). In addition, this dose was found to overlap with both low and high doses (Fig. 3). Therefore, the medium HC dose was not considered for further comparisons and in subsequent analyses and comparisons between low HC vs high HC were used to determine how HC dose affects the human plasma metabolome.

In a comparison of low HC vs high HC, 23 putative biomarkers (Table S2) passed the 95% CI filter and showed significant change based on FDR corrected p values, and had an AUC above 0.7. These 23 putative biomarkers were used to rebuild an OPLS-DA model (Figure S3, supplementary) in order to examine its ability to separate observations based on HC dose. The figure shows separation between observations having either low or high HC doses. Approximately 66% of the variability in metabolites was explained by the model, of which 38.5% was due to HC dose alone with the rest being attributable to other factors related to inter-individual variability. The result for the cross-validation of the model is shown in Figure S4. The validity of the number of orthogonal components in the model was examined using an observed versus predicted plot (Figure S4A), the regression line R^2^ = 0.82 indicates a valid model. According to the area under the ROC curve, 23 putative biomarkers that were significantly changed by the HC dose were 100% successful in classifying the observations (Figure S5). There was a significant elevation of branched chain amino acids and their deaminated metabolites after the highest HC dosage (Table S2, supplementary). Purine metabolites, represented by xanthine and hypoxanthine, and C20:4, C22:6 and C18:3 acids, were significantly elevated by the highest HC dose. On the other hand, the steroids pregnenolone sulfate and androsterone glucuronide were the only metabolites that showed significant reduction after the highest HC dose.

**Figure 6:**
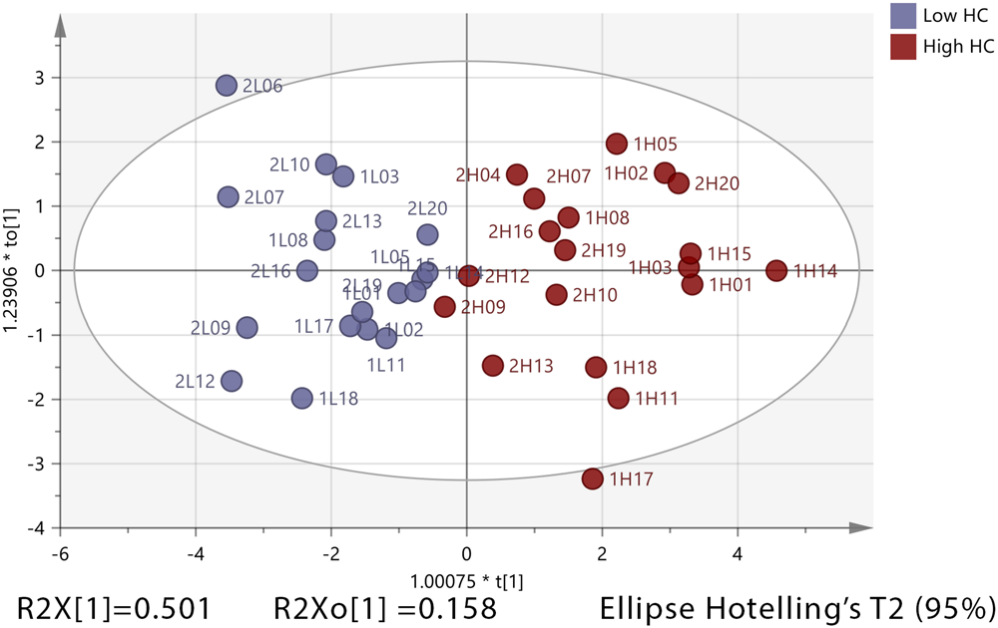
OPLS-DA score plot for the effect of HC dose on 10 significant putative metabolites with highest AUC values in plasma of healthy individuals. The plot shows two groups: subjects with low HC dose (grey-blue) and subjects with high HC dose (red). The model consists of one predictive x-score component; component t[1] and one orthogonal x-score components to[1]. t[1] explains 50% of the predictive variation in x, to[1] explains 15.8% of the orthogonal variation in x, R^2^X (cum) = 0.65.9, R^2^Y (cum) = 1, R^2^ (cum) = 0.74 and accuracy of prediction Q^2^ (cum) = 0.693.

In order to reduce any danger of overfitting a further OPLS-DA model (Fig. 6) was built on the 10 variables having the highest AUC values among the metabolites significantly affected by the HC dose (Table 4) in plasma samples of 38 subjects (low = 19, high = 19). All these metabolites showed varying degrees of positive correlation (0.54 ≤ r ≤ 0.85) except for the steroid conjugates androsterone glucuronide (|r| = 0.7) and pregnenolone sulfate (|r| = 0.85) which were negatively correlated with HC dose. The model shows that about 66% of the variations in these biomarkers were explained by the model, 50% of this variation was due to the HC dose with *P* CV-ANOVA = 4.32E-08, while about 16% was due to orthogonal variation. The area under the ROC indicated an excellent ability of these metabolites to classify 100% of the observations based on HC dose.

**Table 4:** The 10 metabolites with highest AUC values and their correlations (r) to HC dose.

Putative biomarkers	r	99% CI of difference
(S)-3-Methyl-2-oxopentanoic acid^C18^	0.78	(0.159, 0.403)
4-Methyl-2-oxopentanoate*	0.85	(0.193, 0.418)
L-Isoleucine*	0.69	(0.118, 0.381)
Methylacetoacetic acid	0.56	(0.021, 0.381)
2-Hydroxybutanoic acid*	0.68	(0.082, 0.408)
2-Ketobutyric acid*	0.54	(0.045, 0.344)
2-Methylbutyroylcarnitine	0.66	(0.04, 0.436)
Xanthine*	0.68	(0.0025, 0.488)
Androsterone glucuronide	−0.7	(−0.418, −0.088)
Pregnenolone sulfate	−0.85	(−0.422, −0.192)

*Retention time confirmed by standard.

### Metabolites affected by both hydrocortisone and insulin

Twelve metabolites were found to be significantly altered by both insulin and HC treatments (p < 0.05) as shown in Table 5.

**Table 5:** Metabolites significantly affected by both interventions.

Putative metabolite	1 L: 1 H: 2 L: 2 H	p-value (HC)	p-value (insulin)
**Fatty acids**			
C20:4	1: 1.34: 0.54: 0.84	0.002	0.002
C22:6	1: 1.44: 0.38: 0.75	0.000289	0.000126
C18:0	1: 1.36: 0.42: 0.71	0.005	0.00002
C17:0	1: 1.37: 0.37: 0.67	0.005	0.000015
C20:0	1: 1.26: 0.41: 0.79	0.001	0.000106
2-Hydroxybutanoic acid*	1: 1.63: 0.62: 1.07	0.000013	0.013
**Branched chain amino acids**			
L-Isoleucine*	1: 1.23: 0.69: 0.98	0.000058	0.000089
L-Leucine*	1: 1.25: 0.77: 0.95	0.002	0.003
L-Valine*	1: 1.18: 0.86: 0.99	0.001	0.021
4-Methyl-2-oxopentanoate*	1: 1.62: 0.62: 1.13	0.0018	0.003
(S)-3-Methyl-2-oxopentanoic acid ^C18^	1: 1.57: 0.71: 1.11	0.000000061	0.001
**Peptide**			
Gamma-Glutamylglutamine	1: 1.19: 0.79: 0.98	0.004	0.017

*Retention time confirmed by standard. ^C18^metabolites identified using C18-AR column, the rest identified using ZICpHILIC column. (in the ratio column, 1 = low insulin, 2 = high insulin, L = low hydrocortisone, H = high hydrocortisone).

All of the 12 metabolites were elevated by HC and reduced by insulin doses respectively. A heat map of the 12 metabolites which were significant in both interventions was plotted using Metaboanalyst based on intensities of each metabolite in each observation (Figure S6). The clearest effect that can be seen in the heat map is that the metabolites reduced in observations with a high dose of insulin plus low dose of HC were elevated in the samples with low insulin plus high HC doses. In addition, individuals responded differently with regard to these metabolites when high insulin and high HC or low insulin and low HC were given.

Based on split-plot ANOVA, only three metabolites (Table 6) showed significant interaction between insulin and hydrocortisone doses. All the three metabolites were significantly increased (p < 0.05) by HC dose but only one of them, 3-methyl-2-oxobutanoic acid, was significantly decreased (p = 0.036) by high insulin dose.

**Table 6:** Metabolites that showed significant interaction in both interventions.

Putative metabolite	Ratio	P-value		
	1 L: 1 H: 2 L: 2 H	Interaction	Hydrocortisone	Insulin
2-methylbutyrylcarnitine	1**:** 1.9**:** 1.07**:** 1.38	0.015	0.000008	0.369
Methylacetoacetic acid	1**:** 1.4**:** 0.94**:** 1.04	0.023	0.000272	0.086
3-Methyl-2-oxobutanoic acid	1**:** 1.3**:** 0.94**:** 1.02	0.014	0.000364	0.036

In the ratio column, 1 = low insulin, 2 = high insulin, L = low HC, H = high HC.

## Discussion

The clearest effect where insulin and hydrocortisone oppose each other is with regard to an effect on the metabolism of branched chain amino acids and their metabolites. High insulin significantly reduces the levels of leucine/isoleucine, valine (branched chain amino acids, BCAs) and their metabolites compared with low insulin (Example chromatograms shown in Figure S7). Increasing the level of HC infusion increases the levels of leucine/isoleucine and their metabolites irrespective of insulin dose. HC in the presence of the low insulin infusion also produces an increase in BCAs as the concentration of HC is increased and this is the strongest metabolic signature of HC action amongst all the significantly altered metabolites. This observation links to the role of BCAs in obesity and insulin resistance observed in the literature^14–16^. Thus, in the current case, a similar effect is observed from a different perspective, where insulin directly lowers BCA levels significantly and HC opposes this effect. HC is known to promote breakdown of muscle proteins^19^, while BCAs are known to promote production of muscle protein^20–22^. Insulin is known to promote production of muscle tissue and this would be consistent with an increased requirement for BCAs and hence a reduction of their circulating levels. BCAs may exert some beneficial effect in the treatment of insulin resistance associated with chronic liver disease. In a rat model with liver cirrhosis, BCAs improved glucose uptake^23^; in rodents, BCAs improve glucose metabolism in hepatocytes, skeletal muscle, and adipocytes^24–26^.

We previously reported that free fatty acids were increased by high cortisol^17^, this current work highlights this effect with regards to poly unsaturated fatty acid (PUFA) metabolism and is in opposition to the effect of insulin. Previously it was observed that omega-3/omega-6 PUFA levels increased in response to BCAs in two cases^26, 27^. In the current case, high insulin lowers the levels of PUFA and, irrespective of insulin, the highest HC dose increases levels of PUFA.

The most comprehensive list of metabolites within a class that were affected by insulin are the free fatty acids (FFAs). Many FFAs are lowered by >  × 2 by insulin infusion and effects are seen on fatty acids with chain lengths between C10 and C22. HC appears to act in an opposite manner to insulin by promoting higher levels of some FFAs in plasma. Presumably a relatively low level of HC is required to increase levels of FFAs and thus increasing the level of the HC infusion does not promote this process any further—a reason why there was no significant difference between medium and high HC doses. Nevertheless, irrespective of insulin, which lowers the levels of FFAs, the effect on some fatty acids of increasing the level of HC in the infusion can be observed.

Batch *et al*. also described the elevation of C3 and C5 acylcarnitines in obese compared with lean subjects and the elevation of these metabolites in rats fed a diet enriched with BCAs^14^. Insulin has a marked effect in lowering acyl carnitines. The most marked effect is in lowering decanoylcarnitine. HC does not have a marked effect on the levels of these metabolites, significantly increases the level of 2-methylbutyrylcarnitine. High HC also promotes a marked elevation of oleoylcarnitine. There is evidence that high levels of long chain fatty acids are toxic, promoting apoptosis via a mechanism involving caspase 2^28^. Carnitine conjugation provides a means removing fatty acids from tissues^29^.

Tryptophan metabolism is regulated by glucocorticoids and insulin which regulate the enzyme tryptophan dioxygenase (TDO)^30–33^. Indolepyruvate as a metabolite in the tryptophan pathway shows significant elevation following high insulin dose and non-significant reduction following HC dose. Bordag *et al*. observed elevation of a number of tryptophan metabolites in plasma following dexamethasone treatment^12^. There is a link between tryptophan metabolism via the kynurenine pathway and purine metabolism. TDO has haem at its active centre and enzyme activity is regenerated by coupling with the superoxide anion. One of the major sources of superoxide in the body is from the action of xanthine oxidise which converts hypoxanthine via xanthine to uric acid^34^. Elevated xanthine and hypoxanthine are associated with the high HC group and this could indicate an increase in xanthine oxidase leading to increased availability of the superoxide required to support TDO activity^33^.

Finally, the other major alterations in response to HC are, as might be expected, in steroid metabolism. HC suppresses ACTH resulting in reduced adrenocortical secretion of precursor steroids which, in the presence of metyrapone, are diverted to adrenal androgens. This likely explains the reduction, with increasing HC, of androsterone glucuronide, a metabolite of adrenal androgens such as dehydroepiandrosterone, dihydrotestosterone or androstenedione; and of pregnenolone sulphate, a metabolic precursor of HC.

Of course, metabolite changes detected in plasma are only an indirect indicator of the biochemical changes in target tissues for cortisol and insulin. The current study is limited to acute manipulations of insulin and cortisol, and may not be replicated with longer term manipulations. In these studies, a ‘pancreatic clamp’ was employed, involving infusion of somatostatin and replacement with glucagon and growth hormone in addition to insulin infusion; different effects of insulin and cortisol might have been observed in the absence of the pancreatic clamp. Moreover, there may be a bias in metabolomic studies in favour of detecting changes in the most abundant, rather than the most biologically important metabolites. Of equal interest, however, is the pragmatic application of changes in metabolites as biomarkers to measure glucocorticoid or insulin action. For insulin, fasting plasma insulin/glucose ratios are the only non-invasive approach to determine insulin sensitivity, and classification of risk of type 2 diabetes could be enhanced by additional biomarkers. By combining 10 markers, we demonstrate high sensitivity to discriminate between high dose and low dose insulin infusion, but further tests in larger numbers will be required to test associations with physiological insulin action. For glucocorticoids, there are no reliable specific or sensitive biomarkers, since even measurement of plasma cortisol is subject to many caveats. We show that a combination of 10 markers has moderately high sensitivity to discriminate between low and high dose HC infusion, although the discrimination of medium dose infusion remained relatively poor (AUC = 0.69). In related work it was found that 7 marker compounds were predictive of corticosteroid dose in patients with congenital adrenal hyperplasia undergoing hormone replacement therapy (paper submitted). It remains to be tested whether the markers discovered in the current study, in combination or alone, will be sensitive to physiological or pharmacological variation in cortisol action, and crucially whether they have specificity when compared with effects of obesity and other features which accompany glucocorticoid excess.

## Materials and Methods

### Chemicals and materials

HPLC grade acetonitrile (ACN) was purchased from Fisher Scientific, UK. HPLC grade water was produced by a Direct-Q 3 Ultrapure Water System from Millipore, UK. AnalaR grade formic acid (98%) was obtained from BDH-Merck, UK. Ammonium carbonate and ammonium acetate were purchased from Sigma-Aldrich, UK.

### Sample collection

The study protocol has been described in detail previously^17^. In brief, 20 healthy men (age 33.4 ± 3.5 years, BMI 23.8 ± 03 kg/m^2^) were recruited to a randomised crossover study. Written informed consent was obtained from each participant as well as approval from the South East Scotland Research Ethics Committee (reference number 09/S1102/50). All the experimental protocols were carried out in accordance with the relevant guidelines and regulations concerning the participation of human subjects. Eligibility criteria were as follows: body mass index 20–25 kg/m^2^; normal screening blood tests (full blood count, glucose, liver, renal and thyroid function); alcohol intake ≤ 21 units/week; no medical conditions or on any regular medications; no glucocorticoid therapy by any route in the previous 12 months; weight of of < 5% in the past 6 months. Subjects attended for three study days after overnight fast. Subjects were randomised to receive either low dose (0.06 mU/kg/min) or medium dose (0.2 mU/kg/min) insulin infusion (both groups n = 10) on all three occasions for 6 hours. In addition, intravenous infusions of dextrose, 6,6-^2^H_2_-glucose, 1,1,2,3,3-^2^H_5_-glycerol, somatostatin, glucagon and growth hormone were commenced. The three study days were separated by at least 3 weeks and comprised, in random order, ‘low’, ‘medium’ and ‘high’ glucocorticoid phases: subjects all received 1 gram of metyrapone orally at 2300 h the night before each assessment and at 0700 h and 1100 h to suppress endogenous adrenal cortisol production; for the low glucocorticoid phase subjects took placebo tablets at 2300 h and 0700 h and were infused with saline to achieve plasma cortisol concentrations of ~150 nM; for the medium glucocorticoid phase, subjects took hydrocortisone 10 mg orally at 2300 h and 5 mg at 0700 h and were infused with hydrocortisone 0.04 mg/kg bolus followed by 0.025 mg/kg/h to achieve plasma cortisol levels of ~400 nM; for the high glucocorticoid phase subjects took hydrocortisone 20 mg orally at 2300 h and 10 mg at 0700 h and were infused with hydrocortisone 0.18 mg/kg/h bolus and 0.12 mg/kg/h to achieve plasma cortisol concentrations of ~1400 nM^17^. Samples for metabolomics analysis were obtained after 4 hours of the protocol.

### Sample preparation

Plasma samples were stored at −30 °C and thawed at room temperature prior to preparation for LC-MS analysis. For analysis using ZIC-pHILIC conditions, 200 µl of plasma was thoroughly mixed with 800 µl of acetonitrile, followed by centrifugation at 3000 revolutions per minute (RPM) for 5 minutes; 800 µl of supernatant was then transferred to a LC vial. For the RP conditions, 200 µl of plasma was diluted with 800 µl of acetonitrile and followed by centrifugation at 3000 revolutions per minute (RPM) for 5 minutes.

### LC-MS data acquisition

Samples were randomly placed in the autosampler tray and the LC-MS experiment was performed on an Accela 600 HPLC system combined with an Exactive (Orbitrap) mass spectrometer from Thermo Fisher Scientific (Hemel Hempstead, UK). In separate runs, 10 μL of sample was injected onto two columns obtained from HiChrom Ltd., Reading, UK: a ZIC-pHILIC column (150 × 4.6 mm, 5 µm particle) and an ACE C18-AR column (150 × 4.6 mm, 5 μm particles). The LC–MS system was run in binary gradient mode. A flow rate of 0.3 mL/min was used and samples were kept in a vial tray set at 3 °C. The mobile phase conditions were as follows: (i) ZIC-pHILIC: A 20 mM ammonium carbonate pH 9.2 B Acetonitrile; 0 min 80% B 30 min 20% B, 36 min 20% B, 37 min 80% B, 46 min. 80% B. (ii) C18 AR: A 0.1% v/v formic acid in water, B 0.1% v/v formic acid in acetonitrile; 0 min. 5% B 30 min 95% B 36 min 95% B 37 min 5% B 46 min 5% B. The ESI interface was operated in positive and negative ion switching mode, with + 4.0 kV of spray voltage for positive mode and −3.5 kV for negative mode. The temperature of the ion transfer capillary was 270 °C and sheath and auxiliary gas were set at 57 and 17 arbitrary units, respectively. The full scan range of both positive and negative modes was set at 75 to 1200 m/z with AGC target and resolution as Balanced and High (1E6 and 50,000), respectively. Prior to analysis, mass calibration was performed for both ESI modes using the standard Thermo Calmix solution.

The signals at 83.0604 m/z (2xACN + H) and 91.0037 m/z (2 x formate-H) were selected as lock masses for positive and negative mode, respectively. Data were recorded using the Xcalibur 2.1.0 software package (Thermo Fisher Scientific, Hemel Hempstead, UK).

### Data Extraction

Data extraction was carried out using m/z Match software and IDEOM^33^. Metabolites were identified to MSI levels 1 or 2^35^ initially either according to exact mass ( < 3 ppm deviation) plus retention time matching to a standard or according to accurate mass. The extracted data was then modelled as described in supplementary information.

## Electronic supplementary material

Supplementary information.
