# Supplementary material for: Acute interaction between hydrocortisone and insulin alters the plasma metabolome in humans
Source: Sci Rep. 2017 Sep 13;7:11488. doi: 10.1038/s41598-017-10200-9 (PMC5597623; doi:10.1038/s41598-017-10200-9)
Supplement: Supplementary file 1 — Supplementary information. [file 41598_2017_10200_MOESM1_ESM.pdf]

Acute interaction between hydrocortisone and insulin alters the plasma metabolome in humans

Mohammad A. Alwashih[1,3], Roland H. Stimson[2], Ruth Andrew[2], Brian R. Walker[2] and *David G. Watson[1].

[1] Strathclyde Institute of Pharmacy and Biomedical Sciences, University of Strathclyde, Glasgow G4 0RE.

[2]BHF Centre for Cardiovascular Science, Queen's Medical Research Institute, University of Edinburgh, Scotland, UK.

[3] General Directorate of Medical Services, Ministry of Interior, Riyadh 13321, KSA.

Data analysis

Software

All data processing, including data visualisation, biomarker identification, diagnostics and validation was implemented using SIMCA software v.14 (Umetrics AB, Umeå, Sweden) for multivariate analysis. Metaboanalyst 3.0 (www.metaboanalyst.ca)[1] was used to calculate the area under the receiver operating characteristics (ROC) curves for each of the putatively identified biomarkers. In addition, statistical package for social scientists (SPSS) software (version 22.0, IBM SPSS, Chicago, IL) was employed to carry out a split plot analysis of variance (ANOVA) on each of the statistically significant biomarkers by comparing between the

hydrocortisone doses (paired data) and insulin doses (unpaired data) and well as the interaction between the two.

Pre-treatment

Prior to multivariate analysis, data were transformed using (log_2) and Pareto variance scaled (Par) where the responses for each variable are centred by subtracting its mean value and divided by square root of its standard deviation.

Data visualisation and biomarkers identification

Prior to modelling the data, Hotelling's T^2 and DModX limits were employed to detect outliers of samples which could possibly affect the whole model. Samples were removed when above the 99% red line (action limit) of Hotelling's T^2 or exceeded the 95% orange line (warning limit) of Hotelling's T^2 plus Dcrit (critical limit) of DModX[2]. Principal Component Analysis (PCA) is an unsupervised model employed to explore how variables clustered regardless of *Y* class[3]. Orthogonal projections to latent structures (OPLS) is a supervised model that predicts *Y* from *X* and can separate variation in *X* that correlates to *Y* (predictive) and variation in *X* that is uncorrelated to *Y* (orthogonal/systemic). OPLS-DA is a discriminant analysis of OPLS and employed to examine the difference between groups while neglecting the systemic variation. P values of biomarkers were corrected using a false discovery rate (FDR)[4] using Metaboanalyst 3.0 software (http://www.metaboanalyst.ca/). Variable importance in the projection (VIP) was employed, which served to indicate the contribution of each variable in the metabolomic change to a given model compared to the rest of variables[5]; the average VIP is equal to 1. Based on that value, a variable larger than 1 was deemed to have more contribution in explaining *y*[6]. A 95% confidence interval was calculated for each metabolite based on jack-

knifes of uncertainty, which estimated the prediction error rate based on the cross validation rule used[6]. The biomarker selection workflow was as follows:

1. After analysis using SIMCA-P, the metabolites were filtered based on their p-values and 95% CI of mean difference; if a metabolite had a p-value > 0.05 and/or its 95% CI crossed 0, then it was filtered out.
2. All the significant metabolites were processed by using Metaboanalyst in order to get FDR corrected p-values and area under the ROC curves (AUC); if the metabolite had an FDR > 0.05 and/or AUC < 0.7, it was filtered out.
3. The remaining significant metabolites were tested again using split-plot analysis of variance (ANOVA) in order to examine the possibility of significant interaction between both interventions.

Diagnostics and validation

R^2 and Q^2 are diagnostic tools used in both supervised and unsupervised models; R^2 represents the percentage of variation explained by the model, Q^2 indicates the percentage of variation in response to cross validation[7] which meant that it was capable of predicting with a much greater level of accuracy than chance. Cross validation by SIMCA-P - by default - leaves 1/7th of the data out at each iteration, hence, the appropriateness of the cross-validation was examined by a plot of Y observed vs Y predicted; the R^2 value was used to optimise the number of latent variables (orthogonal axis)[8]. A permutations test was applied to supervised models to evaluate whether the specific grouping of the observations in the two designed classes was significantly better than any other random grouping in two arbitrary classes[9]. Model validity was assessed using cross validated ANOVA (CV-ANOVA), corresponding to H_0 hypothesis of equal cross validated predictive residual of the supervised model in comparison

with variation around the mean[10]. The area under curve (AUC) of a receiver operating characteristic (ROC) was used to assess the predictability of the classifier with a rough guide as follows:

0.9–1.0 = excellent, 0.8–0.9 = good, 0.7–0.8 = fair, 0.6–0.7 = poor, 0.5–0.6 = fail (36).

References

1. Xia, J., Sinelnikov, I. V., Han, B. & Wishart, D. S. MetaboAnalyst 3.0--making metabolomics more meaningful. *Nucleic Acids Res* **43**, W251-257, doi:10.1093/nar/gkv380 (2015).
2. Eriksson, L., Byrne, T., Johansson, E., Trygg, J. & Vikstrom, C. in *Multi- and Megavariate Data Analysis* (ed MKS Umetrics) 233-238 (MKS, 2013).
3. Kirwan, G. M. *et al.* Building multivariate systems biology models. *Anal Chem* **84**, 7064-7071, doi:10.1021/ac301269r (2012).
4. Benjamini, Y. & Hochberg, Y. Controlling the False Discovery Rate a Practical and powerful Approach to Multiple Testing. *Journal of the Royal Statistical Society* **57**, 289-300 (1995).
5. Chong, I.-G. & Jun, C.-H. Performance of some variable selection methods when multicollinearity is present. *Chemometrics and Intelligent Laboratory Systems* **78**, 103-112, doi:10.1016/j.chemolab.2004.12.011 (2005).
6. Eriksson, L., Byrne, T., Johansson, E., Trygg, J. & Vikstrom, C. in *Multi- and Megavariate Data Analysis* (ed MKS Umetrics) 233-238 (MKS, 2013).
7. Efron, B. & Gong, G. A Leisurely Look at the Bootstrap, the Jackknife, and Cross-validation. *The American Statistician* **37**, 36-48 (1983).
8. Xi, B., Gu, H., Baniasadi, H. & Raftery, D. Statistical analysis and modeling of mass spectrometry-based metabolomics data. *Methods Mol Biol* **1198**, 333-353, doi:10.1007/978-1-4939-1258-2_22 (2014).
9. Westerhuis, J. A. *et al.* Assessment of PLSDA cross validation. *Metabolomics* **4**, 81-89, doi:10.1007/s11306-007-0099-6 (2008).
10. Eriksson, L., Trygg, J. & Wold, S. CV-ANOVA for significance testing of PLS and OPLS (R) models. *Journal of Chemometrics* **22**, 594-600, doi:10.1002/cem.1187 (2008).

Table S1. Misclassification table showing the proportion of correctly classified observations.

	**Samples (n)**	**Correct**	**LC**	**MC**	**HC**
LC	14	100%	14	0	0
MC	19	52.63%	1	10	8
HC	12	100%	0	0	12
No class	9		3	1	5
Total	54	80%	18	11	25
**Fisher's prob.**	**9.6e-009**				

Table S2. Metabolites that were significantly changed in response to HC dose.

Metabolite	AUC	High HC/Low HC	p-value
**Lipids**			
C22:6	0.72	1.58	0.00028
C20:4	0.74	1.41	0.002
18:0	0.71	1.45	0.005
17:0	0.72	1.49	0.005
20:0	0.73	1.45	0.001
2-Hydroxybutanoic acid *	0.83	1.66	0.00001
2-Oxopentanoic acid	0.72	1.23	0.00004
2-Ketobutyric acid *	0.81	2.02	0.00002
Methylacetoacetic acid	0.80	1.27	0.0002
Androsterone glucuronide	0.84	0.48	3.30E-08
Pregnenolone sulfate	0.95	0.30	1.70E-09
**Branched chain amino acids**			
(S)-3-Hydroxyisobutyrate ^{C18}	0.77	1.52	0.0004
(S)-3-Methyl-2-oxopentanoic acid ^{C18}	0.89	1.56	6.10E-08
4-Methyl-2-oxopentanoate *	0.93	1.69	0.001
L-Leucine *	0.76	1.30	0.002
L-Isoleucine *	0.81	1.24	0.00005
3-Methyl-2-oxobutanoic acid *	0.77	1.25	0.0003
L-Valine *	0.75	1.16	0.001
**Miscellaneous**			
Hypoxanthine *	0.74	1.28	0.004
Xanthine *	0.83	1.30	0.00001

Gamma-Glutamylglutamine	0.75	1.21	0.004
6-methyltetrahydropterin	0.76	1.44	0.008
Valerylcarnitine	0.82	1.58	0.000008

* Confirmed by standard. ^{C18} metabolites identified using C18 column, the rest identified using ZICpHILIC column. (L= low hydrocortisone, H=highest hydrocortisone), p-value obtained from split plot ANOVA.

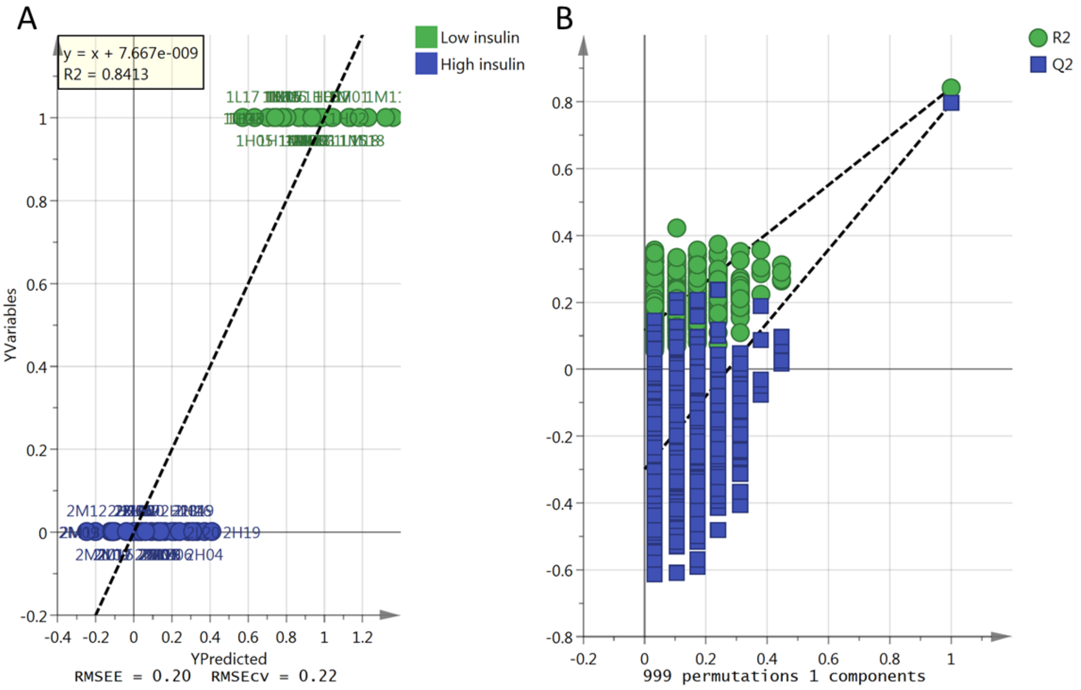

Figure S1. (A) The plot displays the observed (y-axis) versus predicted (x-axis) values of the selected Y-variable of the model. The R^2 of the regression line indicates the goodness of Fit = 0.84, with a good model all the observations will fall close to these 45 degrees of the line, and with less good models the observations are scattered around the regression line. **(B)** Permutations test. The plot shows, for a selected Y-variables, on the vertical axis the values of R^2 and Q^2 for the original model (far to the right) and of the Y-permuted models further to the left. The horizontal axis shows the correlation between the permuted and the original Y. The original Y has the correlation 1.

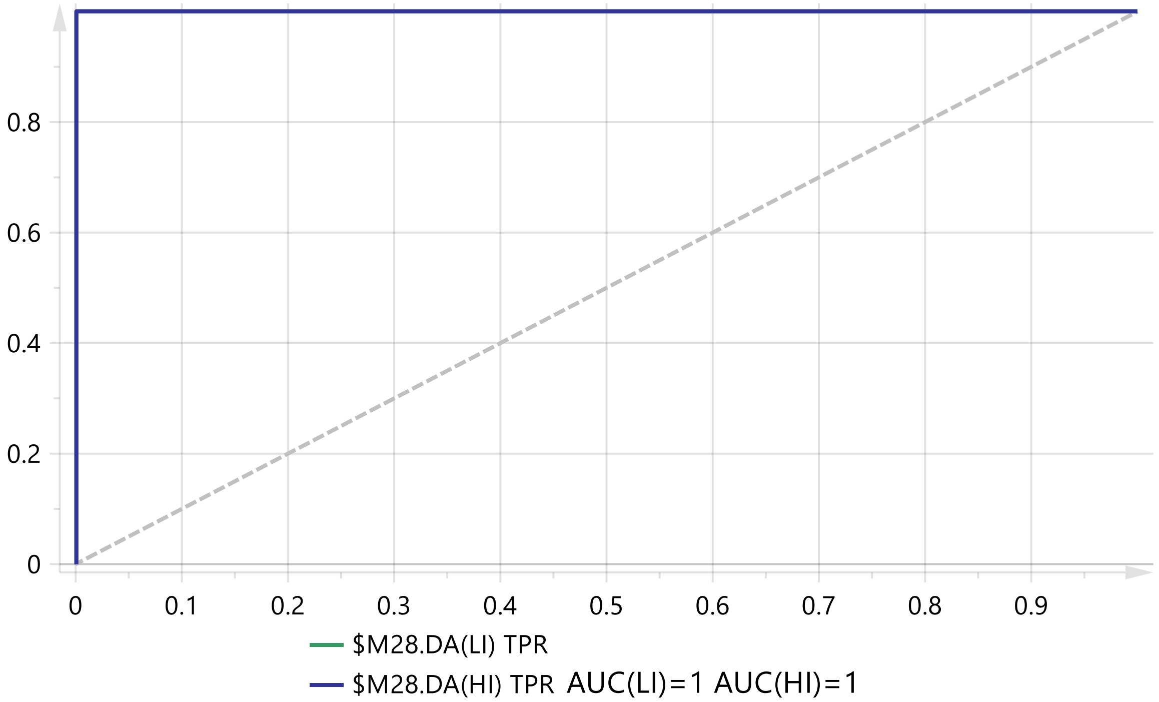

Figure S2. ROC curves show sensitivity (true positive rate (TPR)) on the y-axis versus (false positive rate (FPR = 1 - Specificity)) on the x-axis. The area under the ROC curves (AUC) for the groups; low insulin dose (LI) = 1 and high insulin dose (HI) = 1, based on 29 metabolites.

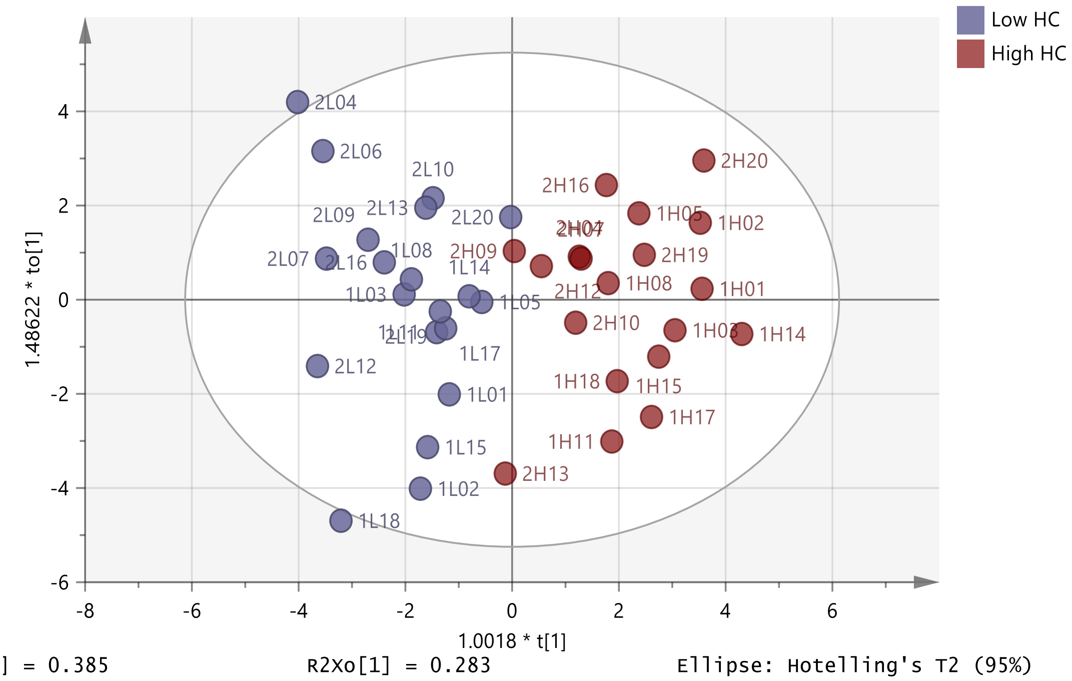

Figure S3. OPLS-DA score plot for the effect of HC dose on 23 significant metabolites in plasma of healthy individuals. The plot shows two groups: low HC dose (grey-blue) and high HC dose (red) observations. The model consists of one predictive x-score component; component t[1] and one orthogonal x-score components to[1]. t[1] explains 38.5% of the predictive variation in x, to[1] explains 28.3% of the orthogonal variation in x, R^2X (cum) = 0.66, R^2Y (cum) = 1, R^2 (cum) = 0.76. Accuracy of prediction Q^2 (cum) = 0.699.

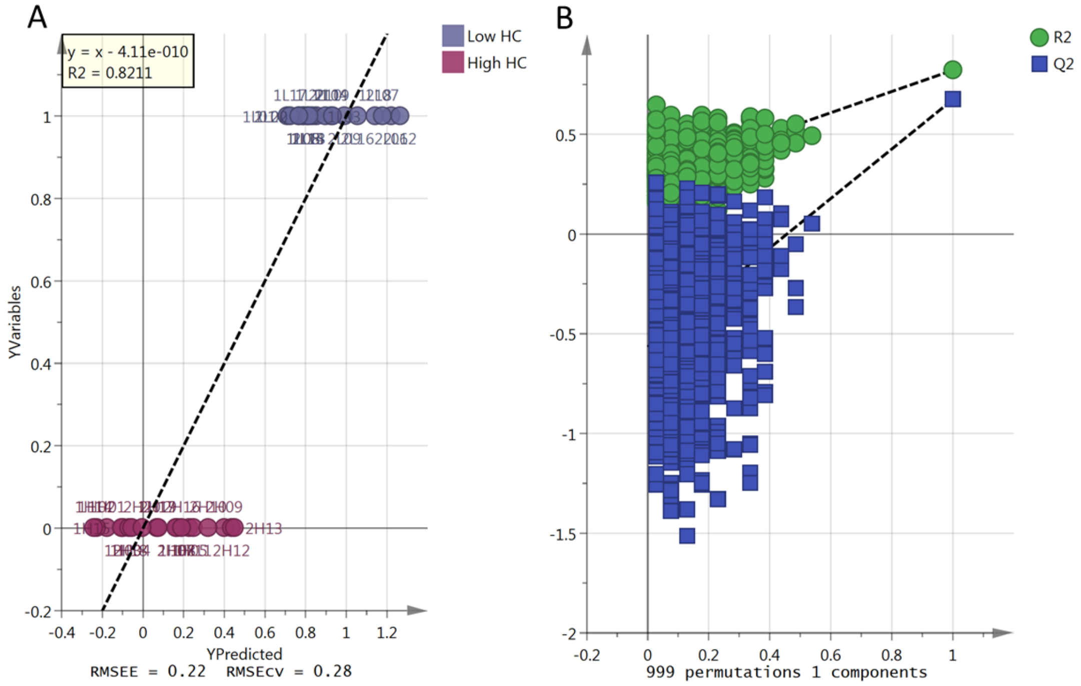

Figure S4. (A) The plot displays the observed (y-axis) versus predicted (x-axis) values of the selected Y-variable of the model. The R^2 of the regression line indicates the goodness of Fit = 0.82, with a good model all the observations will fall close to these 45 degrees of the line, and with less good models the observations are scattered around the regression line. **(B)** Permutations test. The plot shows, for a selected Y-variables, on the vertical axis the values of R^2 and Q^2 for the original model (far to the right) and of the Y-permuted models further to the left. The horizontal axis shows the correlation between the permuted Y-vectors and the original Y-vector for the selected Y. The original Y has the correlation 1.0.

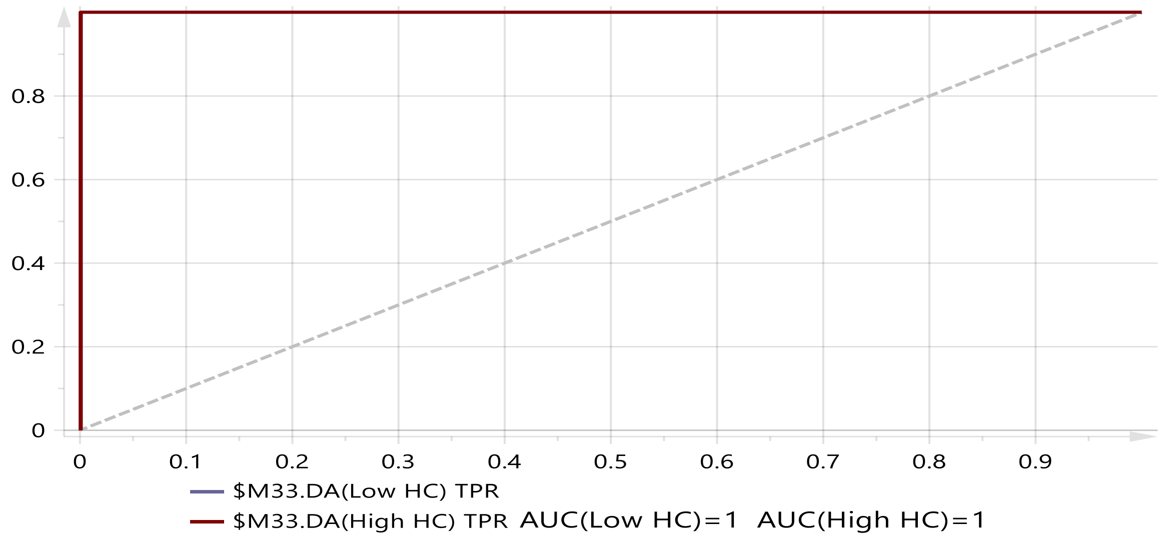

Figure S5. The area under ROC curve shows the ability of the 23 metabolites of separating the two groups. The plot show sensitivity (true positive rate (TPR)) on the y-axis versus (false positive rate (FPR = 1 - Specificity)) on the x-axis. Area under the ROC curves (AUC) for the groups; low HC dose = 1 and high HC dose = 1.

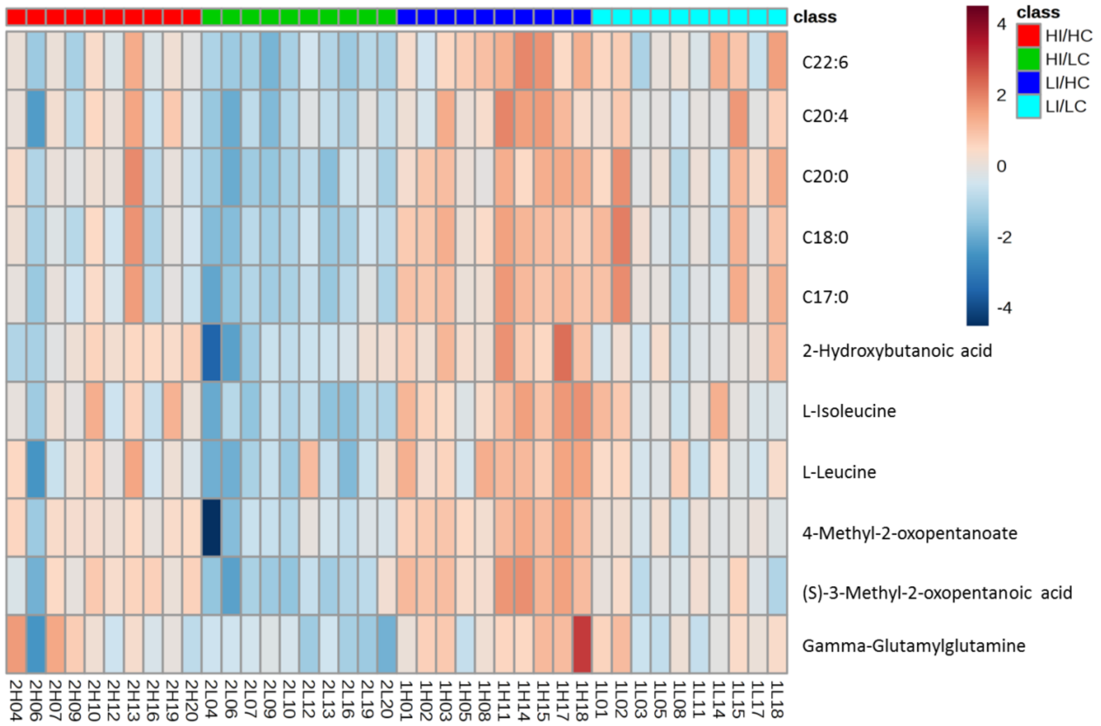

Figure S6. **Heat map shows metabolites that significantly affected by both interventions insulin and HC.** The plot shows heat map of the putative biomarkers (Table 7) that significantly changed following insulin and HC doses in individual samples after excluding medium HC dose (n = 40). The different colour shades represent intensities of each metabolite (rows) in each observation (column). The metabolites were generally increased in observations with low insulin/high HC class (LI/HC - dark blue), decreased in high insulin/low HC class (HI/LC - green), and unevenly disturbed in both low and both high insulin and HC respectively. Valine wasn't given in the heat map from Metaboanalyst.

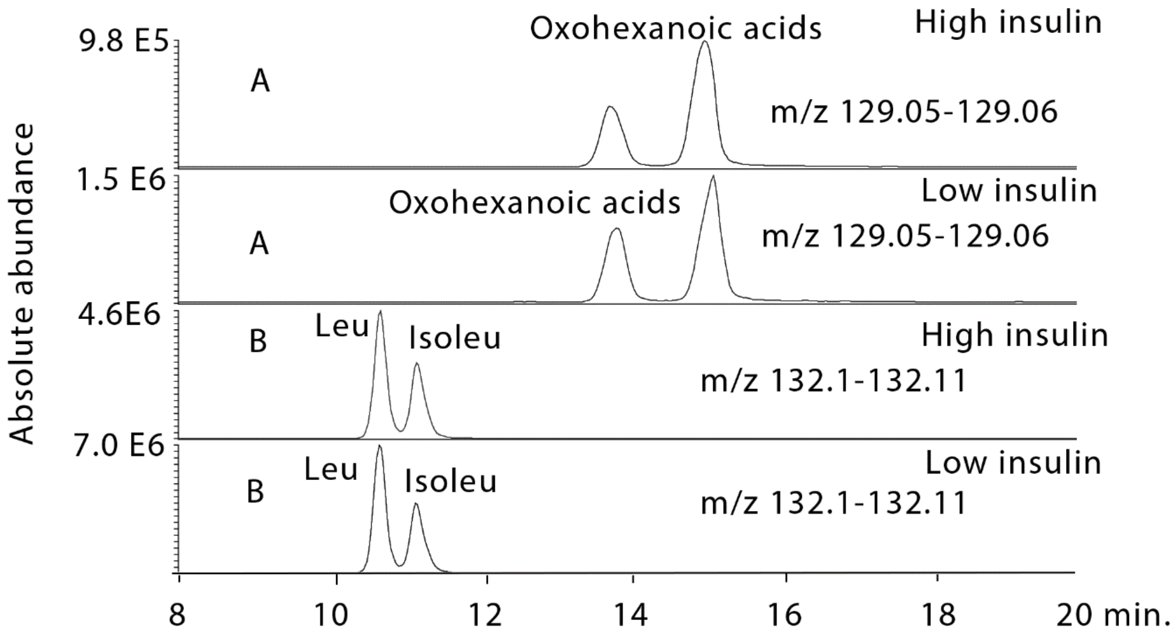

Figure S7. Chromatograms showing the effect of insulin in decreasing the levels of leucine, isoleucine and their oxohexanoic acid metabolites. A Analysis of a C18 column. B Analysis of a ZICpHILIC column.
